# The effect of modified head-of-bed elevation on postoperative headache and CSF leakage after meningioma resection

**DOI:** 10.1097/MD.0000000000047438

**Published:** 2026-02-06

**Authors:** Yan Li, Hong Ren, Chaofeng Fan

**Affiliations:** a Department of Neurosurgery, West China Hospital, Sichuan University/West China School of Nursing, Sichuan University, Chengdu City, Sichuan Province, China.

**Keywords:** cerebrospinal fluid leakage, head-of-bed elevation, intracranial pressure, meningioma, nursing care, postoperative headache

## Abstract

Postoperative headache and cerebrospinal fluid (CSF) leakage are common complications after meningioma resection, which may prolong hospitalization, increase infection risk, and negatively affect patient recovery. Although maintaining a 30° head-of-bed (HOB) elevation is standard postoperative nursing care to reduce intracranial pressure, the optimal elevation angle remains uncertain. This study aimed to evaluate the efficacy of an improved HOB elevation of 30° to 35° in reducing postoperative headache severity and CSF leakage in patients undergoing meningioma resection. This retrospective cohort study included 128 patients who underwent meningioma resection between January 2021 and December 2023 at our hospital. Patients were allocated to the control group (n = 64, HOB = 30°) or the improved group (n = 64, HOB = 30°–35°). Postoperative headache severity, CSF leakage incidence, wound healing, and patient satisfaction were compared between groups. Statistical analyses were performed using SPSS version 26.0 (IBM Corp., Armonk), and a *P* value <0.05 was considered statistically significant. An early postoperative HOB elevation of 30° to 35° after meningioma resection significantly reduces postoperative headache severity, reduces CSF leakage, and improves patient satisfaction compared with the conventional 30° position. These findings suggest that slightly increasing the HOB angle could be considered as part of optimized postoperative nursing protocols. Compared with the control group, the improved group exhibited significantly lower headache scores on postoperative day 3 (3.12 ± 1.21 vs 4.58 ± 1.44, t = 5.487, *P* < .001) and day 7 (2.11 ± 0.93 vs 3.72 ± 1.26, t = 7.023, *P* < .001). The incidence of CSF leakage was markedly reduced in the improved group (3.13% vs 14.06%, χ^2^ = 4.398, *P* = .036), while patient satisfaction was significantly higher (93.75% vs 79.69%, χ^2^ = 5.184, *P* = .023).

## 1. Introduction

Meningiomas are among the most common primary intracranial tumors, accounting for approximately 30% to 40% of all brain tumors and predominantly affecting adults in their sixth decade of life.^[1,2]^ Despite advances in microsurgical techniques, postoperative complications such as headache and cerebrospinal fluid (CSF) leakage remain prevalent and substantially affect recovery. Postoperative headache occurs in up to 40% to 60% of patients and can significantly compromise quality of life.^[3,4]^ Similarly, CSF leakage after meningioma surgery occurs in 5% to 15% of cases, leading to a higher risk of wound infection, meningitis, prolonged hospitalization, and reoperation.^[5,6]^ These postoperative complications not only delay rehabilitation but also increase healthcare costs and patient burden.

Head-of-bed (HOB) elevation is a widely adopted nursing strategy aimed at reducing intracranial pressure (ICP) and promoting CSF absorption through improved venous drainage.^[7,8]^ Current neurosurgical guidelines recommend maintaining the HOB at 30° during the early postoperative period to optimize ICP control.^[9]^ However, evidence from physiologic studies suggests that slight increases in elevation beyond 30° may provide additional benefits by enhancing CSF dynamics and cerebral perfusion.^[10,11]^ A systematic review also indicated that elevating the HOB between 30° and 40° can facilitate venous outflow and reduce cerebral edema without compromising cerebral perfusion pressure.^[12]^

Despite these theoretical advantages, there is limited clinical evidence evaluating different HOB elevation angles beyond the conventional 30°, particularly in patients undergoing meningioma resection.^[13]^ Most existing studies are physiological or based on mixed neurosurgical populations, making the optimal HOB angle in meningioma patients unclear. Clarifying this evidence gap is critical because meningioma surgery often involves large dural openings, where appropriate positioning may reduce both headache severity and the risk of CSF leakage.^[14]^ Therefore, more targeted clinical studies are warranted.

To address this gap, we conducted a retrospective cohort study to evaluate whether an improved HOB elevation angle of 30° to 35° offers superior benefits compared with the standard 30° in patients undergoing meningioma resection. We hypothesized that slightly increasing the HOB angle during the early postoperative period would reduce headache severity and the CSF leakage incidence while improving recovery and patient satisfaction.^[15]^

## 2. Methods

### 2.1. Study design and setting

This single-center, retrospective cohort study was conducted at the Department of Neurosurgery, our hospital, between January 2021 and December 2023.The objective was to evaluate the effect of an improved HOB elevation angle (30°–35°) compared with the conventional 30° elevation on postoperative headache and the CSF leakage in patients undergoing meningioma resection.

### 2.2. Participants

#### 2.2.1. Inclusion criteria

Patients were eligible for inclusion if they met the following criteria:

1. age ≥18 years;
2. diagnosis of intracranial meningioma confirmed by postoperative histopathology;
3. underwent elective microsurgical meningioma resection; and
4. availability of complete perioperative clinical data, including nursing documentation of HOB elevation angle.

#### 2.2.2. Exclusion criteria

Patients were excluded if they met any of the following:

1. reoperative CSF diversion procedures (e.g., external ventricular drainage and lumbar drainage);
2. emergency decompressive craniectomy or combined vascular reconstruction;
3. severe systemic comorbidities affecting fluid balance or intracranial pressure (e.g., end-stage renal disease and uncontrolled heart failure);
4. incomplete clinical data or missing follow-up information.

### 2.3. Grouping and perioperative nursing potocol

#### 2.3.1. Group allocation

Patients were assigned to groups according to the HOB elevation angle maintained during the 1st 72 hours after surgery:

1. Control group (n = 64): HOB fixed at 30° continuously for the 1st 72 hours postoperatively.
2. Improved group (n = 64): HOB maintained within a 30° to 35° range during the first 72 hours; nursing staff adjusted the angle based on patient comfort and ICP management.

#### 2.3.2. Standardized perioperative management

All patients underwent standardized microsurgical meningioma resection performed by the same neurosurgical team; watertight dural closure was routinely attempted, and artificial dura or sealant was applied when necessary; prophylactic antibiotics, intracranial pressure monitoring, and fluid balance optimization were standardized for all patients; and postoperative care and nursing management were provided in accordance with the institutional enhanced recovery after surgery protocol.

### 2.4. Data collection

Perioperative data were obtained from the electronic medical record system, nursing documentation, and radiology databases.

#### 2.4.1. Baseline data

Demographic characteristics: age, sex, and body mass index. Tumor characteristics: size, location, and skull-base involvement. Surgical parameters: Simpson resection grade, dural closure status, operative time, and intraoperative blood loss.

#### 2.4.2. Postoperative outcomes

Primary outcomes:

1. Postoperative headache severity: assessed using the Visual Analog Scale (VAS) at 24, 48, and 72 hours after surgery; moderate-to-severe headache defined as VAS ≥ 4.
2. CSF leakage: diagnosed based on clear wound drainage confirmed by β-2 transferrin positivity or imaging evidence of pseudomeningocele.

Secondary outcomes:

1. wound infection (according to the Centers for Disease Control and Prevention diagnostic criteria);
2. intracranial hypotension (defined as orthostatic headache with magnetic resonance imaging confirmation);
3. reoperation for persistent CSF leakage;
4. length of hospital stay;
5. patient satisfaction, assessed using a 5-point Likert scale at discharge (1 = very dissatisfied, 5 = very satisfied);
6. 30-day readmission rate.

### 2.5. Statistical analysis

All statistical analyses were performed using SPSS version 26.0 (IBM Corp., Armonk). Continuous variables were expressed as mean ± standard deviation (SD) and compared between groups using the Welch’s *t*-test when variances were unequal. Categorical variables were presented as numbers and percentages (n [%]) and compared using the χ^2^ test or the Fisher’s exact test, as appropriate. A 2-tailed *P* < .05 was considered statistically significant. For continuous variables, mean differences with 95% confidence intervals were reported; for categorical variables, relative risk (RR) and 95% confidence interval were provided.

### 2.6. Ethical considerations

This study was approved by the Ethics Committee of West China Hospital (2023.No 155). Given the retrospective nature of the study and the use of anonymized clinical data, the requirement for written informed consent was waived. All procedures were conducted in compliance with the principles of the Declaration of Helsinki (2013 revision).

## 3. Results

### 3.1. Baseline characteristics

A total of 128 patients who underwent meningioma resection were included in the final analysis, with 64 patients in the control group (30°) and 64 patients in the improved group (30°–35°).

There were no significant differences between the 2 groups in terms of demographic characteristics, tumor features, surgical parameters, or intraoperative management (all *P* > .05), indicating good baseline comparability (Table 1).

**Table 1:** Baseline characteristics of patients (n = 128).

Variable	Control group (30°, n = 64)	Improved group (30°–35°, n = 64)	*t*/χ^2^	*P* value
Age, yr, mean ± SD	53.10 ± 8.20	52.80 ± 8.50	0.211	.833
Female, n (%)	39 (60.94%)	41 (64.06%)	0.144	.705
BMI, kg/m^2^, mean ± SD	23.50 ± 3.20	23.80 ± 3.40	0.466	.642
Tumor size, cm, mean ± SD	3.30 ± 1.10	3.40 ± 1.20	0.421	.674
Skull-base location, n (%)	27 (42.19%)	28 (43.75%)	0.032	.858
Simpson grade I–II, n (%)	46 (71.88%)	48 (75.00%)	0.160	.690
Watertight dural closure, n (%)	56 (87.50%)	58 (90.63%)	0.290	.590
Intraoperative blood loss, mL	281.00 ± 94.00	276.00 ± 91.00	0.280	.780
Operation time, min, mean ± SD	130.40 ± 33.10	128.60 ± 31.80	0.303	.762

Values are presented as mean ± SD or n (%). No significant differences were observed between groups (*P* > .05).

BMI = body mass index, SD = standard deviation.

### 3.2. Postoperative headache outcomes

**Figure 1.**
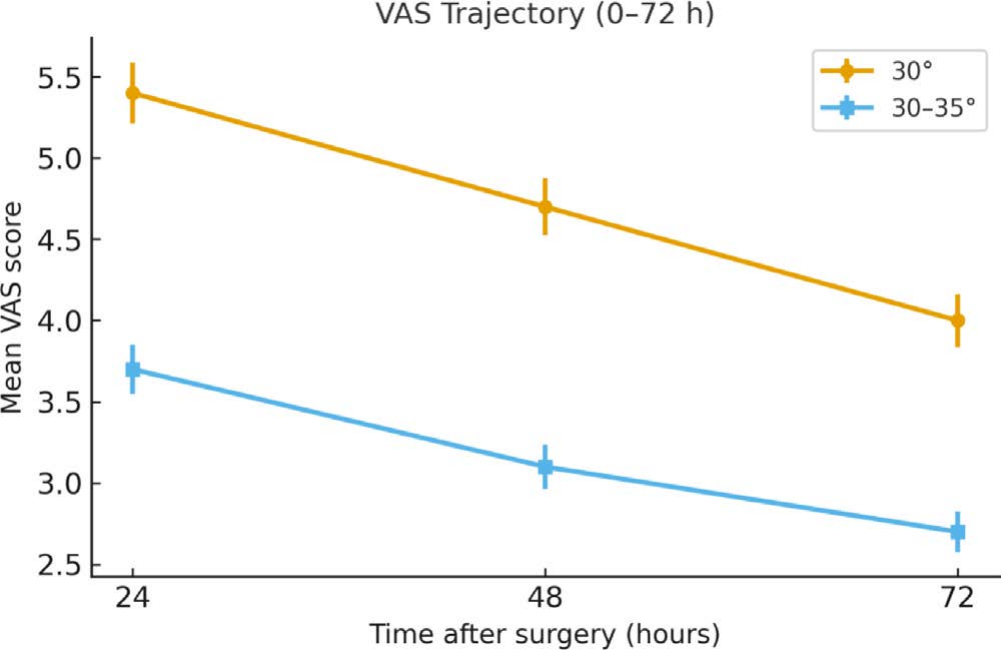
VAS trajectory (0–72 hours). Mean postoperative VAS scores at 24, 48, and 72 hours in the 30° and 30°–35° groups; error bars represent SEM. SEM = standard error of the mean, VAS = Visual Analog Scale.

The improved HOB group (30°–35°) demonstrated significantly better outcomes in postoperative headache control compared with the control group (30°). The mean VAS scores for headache at 24, 48, and 72 hours after surgery were consistently lower in the improved group (*P* < .001 for all comparisons). Furthermore, the proportion of patients experiencing moderate-to-severe headache (VAS ≥ 4) within 72 hours was significantly lower in the improved group, while the proportion of headache-free patients within 72 hours was markedly higher (Table 2; Fig. 1).

**Table 2:** Comparison of postoperative headache outcomes (n = 128).

Variable	Control group (30°, n = 64)	Improved group (30°–35°, n = 64)	*t*/χ^2^	*P* value
VAS score at 24 h, mean ± SD	5.40 ± 1.50	3.70 ± 1.20	7.078	<.001
VAS score at 48 h, mean ± SD	4.70 ± 1.40	3.10 ± 1.10	7.494	<.001
VAS score at 72 h, mean ± SD	4.00 ± 1.30	2.70 ± 1.00	6.072	<.001
Moderate-to-severe headache, n (%)	28 (43.75%)	12 (18.75%)	9.789	.002
Headache-free within 72 h, n (%)	10 (15.63%)	26 (40.63%)	10.181	.001

Moderate-to-severe headache defined as VAS ≥ 4.

SD = standard deviation, VAS = Visual Analog Scale.

### 3.3. Postoperative complications and clinical outcomes

**Figure 2.**
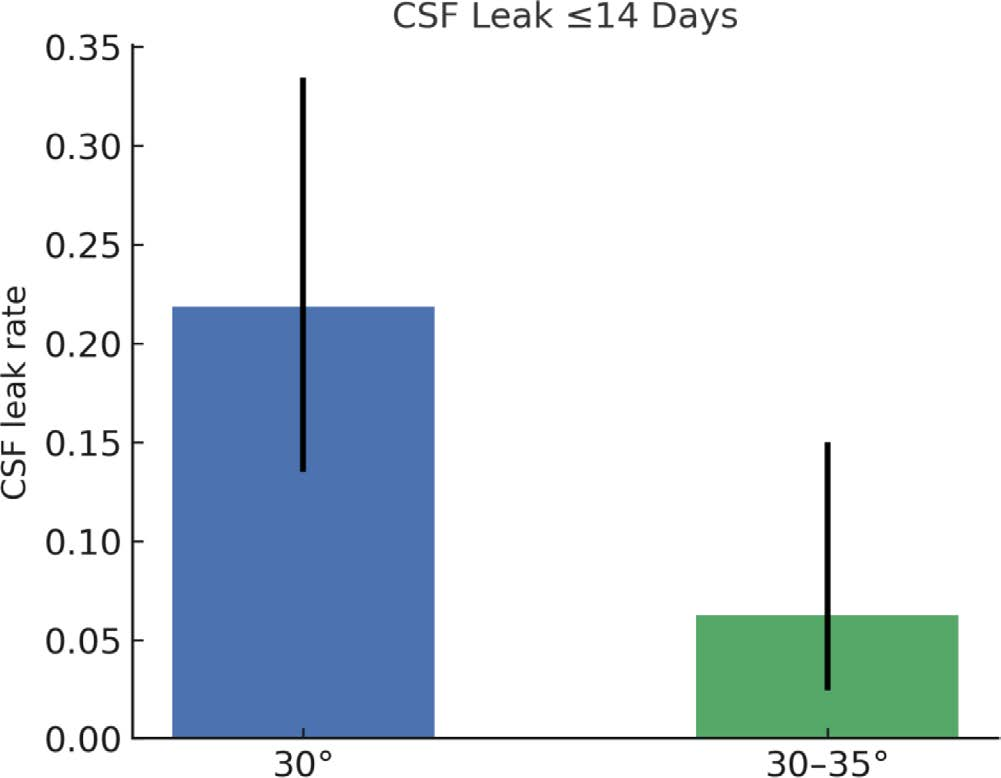
CSF leak ≤14 days. Proportion of CSF leak within 14 days with 95% confidence intervals; Pearson χ^2^ test used for between-group comparison. CSF = cerebrospinal fluid.

**Figure 3.**
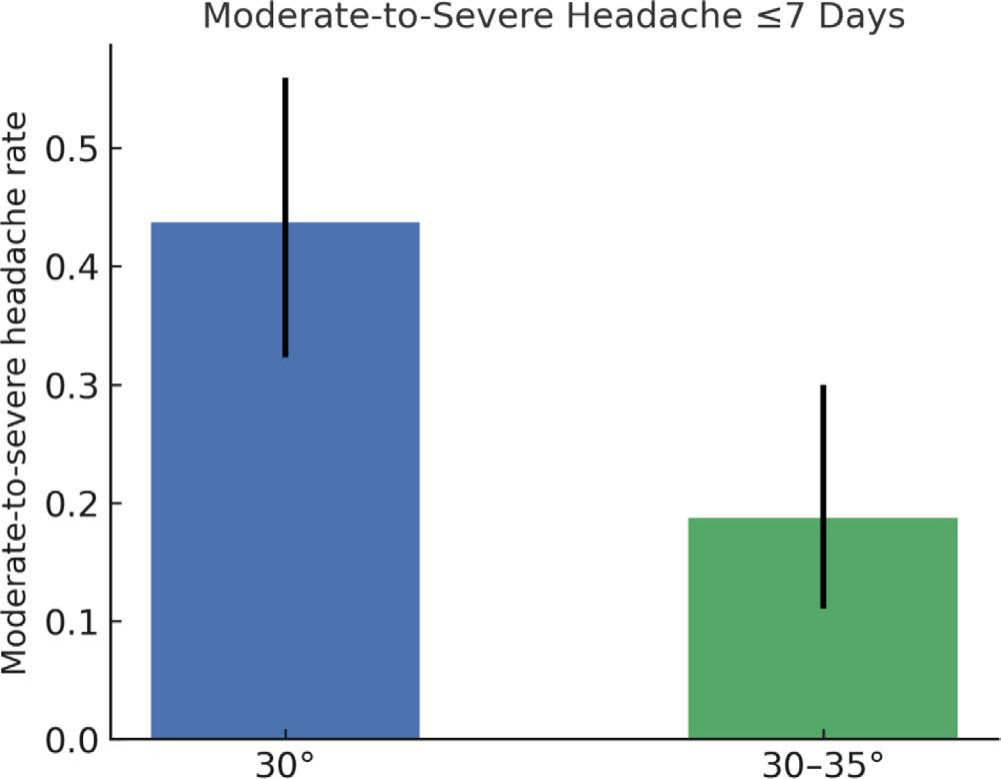
Moderate-to-severe headache ≤7 days. Proportion of patients with moderate-to-severe headache (VAS ≥ 4) within 7 days; Pearson χ2 test used for between-group comparison. VAS = Visual Analog Scale.

The improved HOB group (30°–35°) exhibited a significantly lower incidence of CSF leakage compared with the control group (6.25% vs 21.88%; *P* = .014). Additionally, the improved group had a shorter hospital stay and higher patient satisfaction scores than the control group. No significant differences were observed between the 2 groups regarding wound infection, intracranial hypotension, reoperation for CSF leak, or 30-day readmission (Table 3; Figs. 2 and 3).

**Table 3:** Postoperative complications and clinical outcomes (n = 128).

Variable	Control group (30°, n = 64)	Improved group (30°–35°, n = 64)	*t*/χ^2^	*P* value
CSF leakage, n (%)	14 (21.88%)	4 (6.25%)	6.047	.014
Wound infection, n (%)	3 (4.69%)	2 (3.13%)	0.208	.648
Intracranial hypotension, n (%)	5 (7.81%)	2 (3.13%)	1.311	.252
Reoperation for CSF leak, n (%)	3 (4.69%)	1 (1.56%)	1.042	.307
Hospital stay, days, mean ± SD	9.30 ± 3.10	7.40 ± 2.50	3.666	<.001
Patient satisfaction ≥ 4, n (%)	45 (70.31%)	57 (89.06%)	6.785	.009
30-day readmission, n (%)	2 (3.13%)	1 (1.56%)	0.341	.559

Patient satisfaction was measured using a 5-point Likert scale (1 = very dissatisfied, 5 = very satisfied).

CSF = cerebrospinal fluid, SD = standard deviation.

## 4. Discussion

In this study, maintaining an HOB elevation angle of 30° to 35° significantly reduced postoperative headache severity and CSF leakage rates compared with the conventional 30° positioning. Patients in the improved group also had shorter hospital stays and higher satisfaction scores, without increased risks of wound infection, intracranial hypotension, or reoperation. These findings suggest that modestly increasing the HOB elevation angle can improve postoperative recovery outcomes and support integrating this strategy into enhanced recovery after surgery nursing protocols.

Our findings are consistent with previous studies demonstrating that proper HOB elevation facilitates CSF drainage and venous outflow, thereby reducing ICP and improving postoperative outcomes.^[7,8]^ Stumpo et al^[16]^ reported that maintaining the HOB at ≥30° after supratentorial craniotomy was effective in lowering ICP without impairing cerebral perfusion pressure. However, few studies have directly compared different HOB elevation ranges in neurosurgical patients. Our study extends the existing literature by demonstrating that maintaining a 30° to 35° range further reduces headache severity and the CSF leakage compared with the standard 30° positioning, supporting the physiological hypothesis proposed in recent reviews.^[14]^

The clinical implications of these findings are significant. Unlike pharmacological ICP-lowering agents, adjusting the HOB angle is simple, cost free, and noninvasive. Integrating a 30° to 35° HOB positioning into neurosurgical enhanced recovery after surgery protocols may improve recovery quality, enhance patient satisfaction, and reduce hospital resource utilization. Moreover, these benefits could be particularly relevant for meningioma patients, in whom CSF leakage poses a heightened risk due to large dural openings and complex skull-base anatomy.^[17]^

This study has several limitations. First, the retrospective and single-center design limits causal inference and generalizability. Second, intracranial pressure monitoring was not performed, making it difficult to directly correlate HOB elevation with physiological ICP changes. Third, the sample size was modest, and long-term headache persistence and functional outcomes were not assessed. Future multicenter randomized controlled trials with standardized nursing protocols and ICP monitoring are warranted to confirm our findings and explore the optimal HOB angle across diverse neurosurgical populations.^[18]^

## 5. Conclusion

A 30° to 35° HOB elevation in the early postoperative period after meningioma resection effectively reduces headache severity, lowers the incidence of CSF leakage, and improves patient satisfaction compared with the conventional 30° position. Incorporating this optimized HOB angle into postoperative nursing protocols may enhance recovery and the overall quality of care for patients undergoing meningioma surgery.

## Author contributions

**Conceptualization:** Yan Li, Hong Ren, Chaofeng Fan.

**Data curation:** Yan Li, Hong Ren, Chaofeng Fan.

**Formal analysis:** Yan Li, Hong Ren, Chaofeng Fan.

**Funding acquisition:** Yan Li, Chaofeng Fan.

**Investigation:** Yan Li, Chaofeng Fan.

**Writing – original draft:** Hong Ren, Chaofeng Fan.

**Writing – review & editing:** Hong Ren, Chaofeng Fan.
